# EXOSC5 promotes proliferation of gastric cancer through regulating AKT/STAT3 signaling pathways

**DOI:** 10.7150/jca.69166

**Published:** 2022-02-28

**Authors:** Xiangliu Chen, Yingying Huang, Jin Liu, Wu Lin, Chuanzhi Chen, Yiran Chen, Yongfeng Ding, Yan Yang, Yanyan Chen, Haiyong Wang, Lisong Teng

**Affiliations:** Department of Surgical Oncology, The First Affiliated Hospital, School of Medicine, Zhejiang University.

**Keywords:** EXOSC5, gastric cancer, proliferation, AKT, STAT3, Prognosis

## Abstract

**Purpose:** Exosome component 5 (EXOSC5) is a non-catalytic component of the RNA exosome complex, which is interacted with the Zinc-finger antiviral protein to degrade the target RNA and aberrantly expressed in various malignances. We explored the molecular mechanisms and biological roles by which EXOSC5 promotes the progression of GC.

**Methods:** We used quantitative real-time PCR, Western blotting and immunohistochemistry to analyze EXOSC5 expression in GC samples. An GC organoid-based functional model was assessed, and cancer cell CCK-8 assay, colony formation assay and flow cytometry were performed to reveal the role of EXOSC5 in GC cell proliferation and tumorigenesis. *In vivo*, nude mice tumorigenesis assay were performed to explore the effects of EXOSC5 knockdown on growth of GC. The roles of EXOSC5 on AKT and STAT3 signaling pathways were measured by Western blot.

**Results:** The expression of EXOSC5 was up-regulated in GC tissues and cell lines compared with normal group, and highly expressed EXOSC5 indicated a poorer clinical outcome for GC patients and was positively correlated with tumor size and TNM stage. EXOSC5 overexpression facilitated the growth of GC cells and organoids, while EXOSC5 downregulation inhibited proliferation and induced G1/S phase transition arrest. Moreover, mechanistic studies demonstrated that EXOSC5 increased cyclinD1 expression levels and decreasing the expression levels of p21 and p27 via regulation of the AKT and STAT3 pathway.

**Conclusion:** The expression of EXOSC5 is upregulated and correlated with tumorigenesis and poor prognosis of GC. EXOSC5 increases GC proliferation partly through activating AKT and STAT3 pathways.

## Introduction

Gastric cancer (GC) is the second commonest cause of cancer related death among both men and women worldwide 1. Although the incidence of GC has declined gently for recent two decades, it still has been associated with high level of mortality in Eastern Asia (especially in Japan, China and Korea) 2. It is particularly severe that the overall 5-year survival rate is only nearly 30% due to poor prognosis in the most countries 3. Nevertheless, the molecular mechanisms involved in the development and progression of GC remain poorly understood. It is so urgent for researchers to explore the unknown pathogenesis of gastric carcinogenesis and identify novel diagnostic biomarkers and promising therapeutic targets.

The exosome complex is a ribonuclease complex composed of ten evolutionarily conserved subunits capable of processing and degrading multiple classes of RNA molecules both in the nucleus and cytoplasm 4. In humans, the EXOSC4-9 subunits and their orthologs generate a central barrel-shaped core, which is covered by a three-subunit cap (EXOSC1-3). Exonucleases (DIS3, DIS3L, EXOSC10) involved in the processing and decay of cellular RNAs are bind to the catalytically inactive core and cap subunits of the exosome complex 5. EXOSC5 is one of non-catalytic subunits in the RNA exosome complex, which could be recruited by and directly bound to the Zinc-finger antiviral protein to degrade the target RNA 6. In addition, studies have shown that EXOSC5 is associated with arrhythmia 7, neurodevelopmental disorder 8 and carcinogenesis 9-11. Although, emerging evidence suggests a close association between EXOSC5 and the progression of various cancer, we still know little about it. Most studies have focused on its roles in hematological tumors, and showed that EXOSC5 was upregulated and acted as a cancer-specific immunotherapy target in hematological tumors 12. Recently, pan et al. found that EXOSC5 could promote proliferation of colorectal cancer via activation of ERK and AKT signaling pathways 10. Taken together, EXOSC5 appears to play a vital part in the progression of cancer, which encouraged us to explore whether EXOSC5 is implicated in the oncogenesis of gastric cancer.

In this study, the expression profile and clinical features of EXOSC5 and its correlations with GC patients were explored by analyzing several public databases and clinical samples. We demonstrated that EXOSC5 contributed to the growth of GC and alters the cell cycle distributions. EXOSC5 can promote the proliferation in GC, at least partially, by activating AKT and STAT3 signaling pathways, thus leading to regulate the expression of cell-cycle regulator p21, p27 and cyclin D1 in GC cells and organoids. Importantly, this is the first study reporting the oncogenic and prognostic roles of EXOSC5 in GC.

## Materials and methods

### Tissue samples and cell lines

A total of primary GC and matched normal tissue specimens (Zhejiang cohort 1 N=43 for qPCR; Zhejiang cohort 2 N=10 for Western blot; Zhejiang cohort 3 N=130 for IHC) were obtained from the First Affiliated Hospital of Zhejiang university from 2015 to 2016. All patients had not received preoperative radiotherapy and chemotherapy before surgery, and underwent pathological diagnosis by two professional pathologists according to the criteria of the American Joint Committee on Cancer (AJCC) TNM classification.

Five human GC cell lines (AGS, HGC27, MGC803, MKN45, SNU1) and the immortal normal gastric mucosa epithelium cell line (GES-1) were purchased from the Cell Bank of Chinese Academy of Sciences (Shanghai, P.R. China). GC cell lines were cultured in the Roswell Park Memorial Institute (RPMI)-1640 medium with 10% feta bovine serum (FBS, Gibco, USA), and GES-1 was grown in Dulbecco's modified Eagle's medium (DMEM, Gibco, USA) with 10% FBS, maintained at 37 °C incubator in a 5% CO2 humidified atmosphere consisting.

### Immunohistochemistry (IHC)

Immunohistochemistry was performed as described previously 13. After deparaffinization, all sections were deparaffinized with xylene and dehydrated with gradient alcohol followed by inactivation of endogenous peroxidase activity by 3% H_2_O_2_ in methanol for 10 mins. Non-specific binding was blocked by incubation with 10% normal goat serum in phosphate-buffered saline (PBS) for 1 hour at room temperature. The slides were incubated with primary antibody EXOSC5 (Proteintech, 15627-1-AP) overnight at 4 °C, followed by biotinylated goat anti-rabbit lgG (Sigma, MO, USA) for 1 hour at room temperature. Then, a streptavidin-biotin-peroxidase complex assay was carried out. The peroxidase activity was developed by incubating with 0.1% 3,3-diaminobenzidine (Sigma) in PBS with 0.05% H_2_O_2_ for 5 min at room temperature. Based on the frequency and intensity of staining, the positive rate was scored as 0 (no staining), 1 (1-25%), 2 (26-50%), 3 (51-75%) or 4 (76-100%), and the staining intensity was graded as 0 (negative), 1 (weak), 2 (intermediate) or 3 (strong).

### RNA Extraction and qRT-PCR Assay

TRIzol reagent (Invitrogen, Carlsbad, California, USA) was used for the extraction of the total RNA from frozen tissues and cultured cells. PrimeScript RT Reagent Kit (Takara, Shiga, Japan) was used to synthesize for complementary DNA (cDNA). SYBR Green Premix Ex Taq (Takara, Shiga, Japan) on an ABI 7500 PCR system (Applied Biosystems, CA, USA) was used to carry out qRT-PCR, according to the standard quantitative PCR program. The primer sequences used were: EXOSC5 forward: 5'- ACTTTGCCTGCGAACAGAACC-3′, EXOSC5 reverse: 5′-CTCTTTGCTGACCTTCACCTC-3′; GAPDH forward: 5′-GGAGCGAGATCCCTCCAAAAT-3′, and ′′GAPDH reverse: 5- GGCTGTTGTCATACTTCTCATGG-3. Relative expression of EXOSC5 was calculated by 2-ΔΔCt method according to the internal control GAPDH.

### Protein Extraction and Western blotting

The total proteins of frozen tissues and cultured cells were extracted in RIPA buffer (Beyotime Inc., Chian) with phosphatase inhibitor and protease inhibitor (Beyotime Inc., Chian). The Cell lysates were centrifuged at 1.2 × 10^4^ g, 4 °C for 20 min. The concentrations of the proteins were detected using the BCA Protein assay kit (Thermo Scientific, USA). Identical quantities of proteins were separated by SDS-PAGE, and transferred onto PVDF membranes (Millipore, USA). After that, membranes were incubated with primary antibodies at 4 °C overnight. Then, the membrane was washed with TBST three times and incubated with an HRP-conjugated secondary antibody (Cell Signaling Technology, USA). GAPDH (Cell Signaling Technology, USA) was used as standard loading control. The primary antibodies are as following: EXOSC5 (Proteintech, 15627-1-AP); p21 (Proteintech, 10355-1-AP); p27 (Proteintech, 25614-1-AP); cyclin D1 (CST, 2922S); p-AKT (CST, 9271S); AKT (CST, 9272S); p-STAT3 (CST, 9145); AKT (CST, 9272S).

### Lentivirus packaging and Transfection

EXOSC5 short hairpin RNAs (shEXOSC5), EXOSC5 overexpression plasmid and their control were purchased from GeneRay Company (Shanghai, China). The target sequences are: shRNA1#1, 5′- GAAGGTCAGCAAAGAGATT-3′; shRNA#2, 5′- CGAAGTGATCCTGAGGCCGAAGATT-3′. For lentiviral packaging, HEK293T cells (1.5 ×10^5^) were seeded in 6-well plates, incubated for 24 h and then transfected with 1μg of plasmids and 1μg Lentivirus Package plasmid mix diluted in 200 μl Opti-MEM (Gibco, USA) with 4 μl Lipofectamine 3000 (Invitrogen, L3000015) according to this protocol. At 48 h and 72 h, the virus-containing medium was harvested for the first and second time, and then transferred through 0.45 μm filter. Next, the medium were centrifuged at 2×10^4^ g for 2 hour. The precipitate were suspended in 50 μl PBS and stored at -80 °C. For lentivirus infections, the AGS and HGC-27 cells were cultured in 24-well plates for infection by virus-containing PBS with polybrene (Genechem, REVG0001), incubated at 37 °C for 24 h and replaced by 1640 medium with 10% FBS. For overexpression plasmid transfection, MGC-803 and MKN45 were transfected with EXOSC5 overexpression plasmids using Lipofectamine 3000 following the manufacturer's protocol.

### Cell Growth Assay

For the CCK-8 assay, 2000 cells were trypsinized and inoculated in 96 well plate. Then, 10 μL of CCK-8 (Dojindo, Kumamoto, Japan) solution was added to each well and absorbance at 450 nm was measured after 2 hour of incubation once a day for 5 days. For colony formation assay is as follows: 1000 cells were seeded in 6-well plate and cultured for 10 days. Then cell colonies were fixed using 4% paraformaldehyde, and stained with 0.1% crystal violet (Sigma, St. Louis, MO). The number and size of colonies with more than 50 cells was detected. All experiments were performed in triplicate.

### GC organoid model

Human GC organoids were cultured as described previously to simulate the tumor microenvironment of the human body 14. A EXOSC5 overexpression plasmid and two EXOSC5 shRNA were transfected into the GC organoids to investigate and verify the role played by EXOSC5 in human gastric cancer progression. The growth of GC organoids was observed daily by microscope.

### Flow Cytometric Analysis of Cell Cycle

The AGS and HGC-27 cells were seeded into 6-well culture plate with 2 × 10^5^ cells per well. After 48 h, the cells were collected, centrifuged and rinsed with precooling PBS twice. Then the cells were fixed with 75% ethanol at 4 °C overnight or -20 °C for long-term storage. Finally, the cells were centrifuged and added with 500 μl DNA staining solution (Multi Sciences, China) to stain the DNA. The stained cells were evaluated by flow cytometry (BD Calibur, San Jose, CA) and the data was analyzed by FlowJo 10.1.

### *In vivo* xenograft models

Female nude mice (BALB/C, 4-5 weeks) were purchased from Hangzhou Ziyuan Laboratory Animal Technology Company. All animal experiments were approved by the Institutional Animal Care and Use Committee at the First Affiliated Hospital of Zhejiang university. For xenograft models, 5 × 10^6^ HGC-27 stably transfected with shRNA-EXOSC5 or shRNA-NC injected subcutaneously in the right flank of mice (four mice per group). Tumor volume was measured every 5 days, and tumor weight was weighed at the end of the experiment.

### Statistical analysis

T-test and one-way ANOVA was performed to compare the differences between groups. The correlations between IHC scores and clinicopathologic characteristics were using chi-square test and Logistic multivariate analysis. Survival rates were calculated using Kaplan-Meier method. Cox regression model was used to identify independent prognostic factors associated with overall survival. P value < 0.05 was considered statistically significant. All statistical analyses were performed by SPSS version 22.0 (IBM, USA).

## Results

### EXOSC5 is upregulated and correlated with poor prognosis in GC

**Figure 1:**
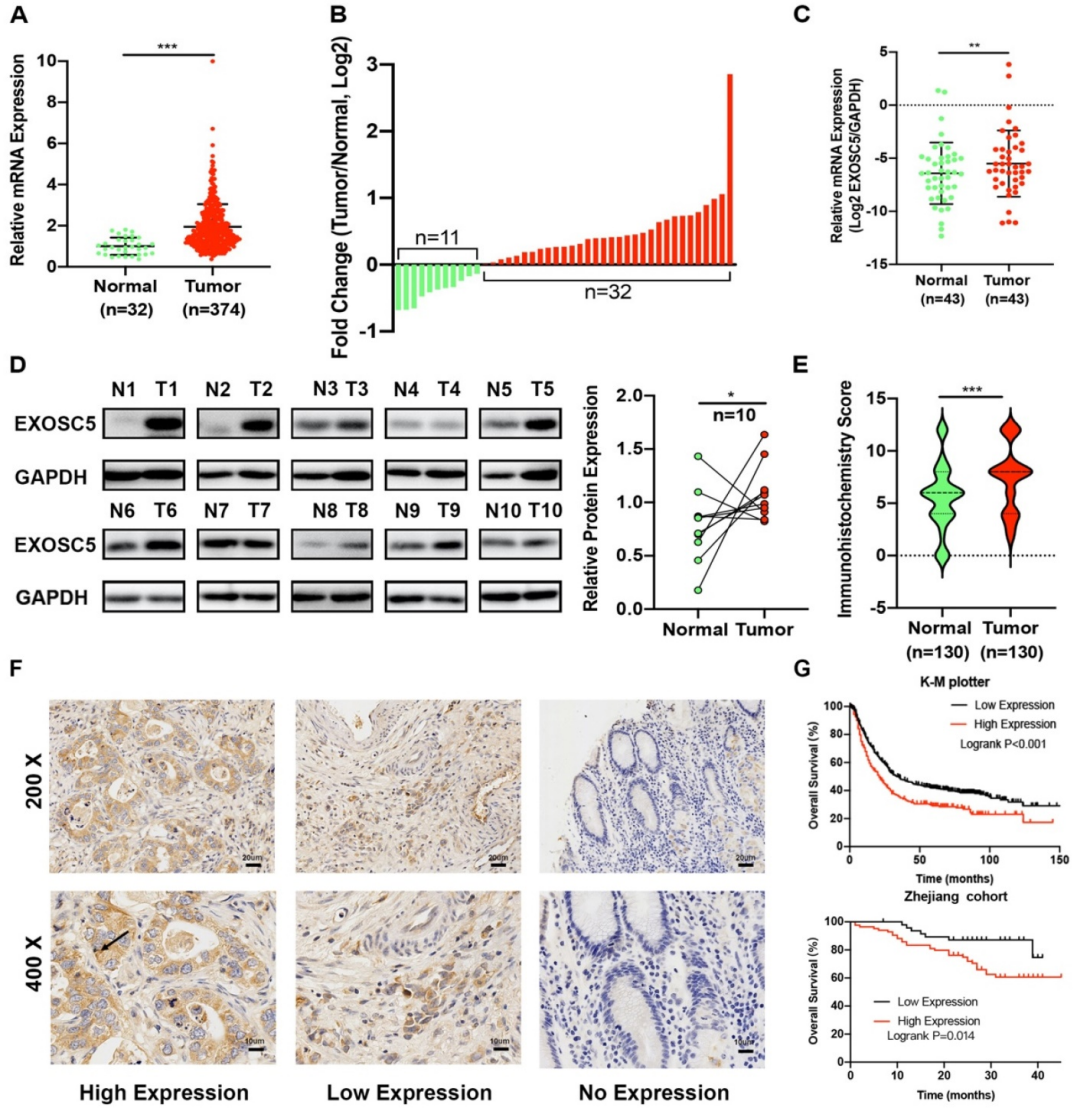
** EXOSC5 was overexpressed in CG tissues, and correlated with overall survival. (A)** EXOSC5 mRNA level in GC and normal gastric tissues in TCGA database. **(B, C)** EXOSC5 mRNA levels in GC and normal gastric tissues in Zhejiang cohort 1. **(D)** Representative images of EXOSC5 proteins level in Zhejiang cohort 2. **(E, F)** IHC analysis of EXOSC5 in Zhejiang cohort 3. **(G)** The association between EXOSC5 expression and overall survival (OS) in GC patients assessed by K-M plotter and Zhejiang cohort 3. Data are shown as the mean ± standard deviation. *P < 0.05, **P < 0.01, ***P < 0.001.

First, we analyzed the mRNA expression of EXOSC5 in GC and normal tissues using the Cancer Genome Atlas (TCGA). The result revealed that EXOSC5 was remarkably up-regulated in GC tissues compared to normal tissues (Figure 1A). In order to verify this data, we elucidated EXOSC5 mRNA expression in Zhejiang cohort 1 (43 matched GC and adjacent non-tumor samples). EXOSC5 expression level was similarly increased in 74.41% (32/43) of CG tissues as compared to that in normal tissues (Figure 1B, 1C). Then the high expression of EXOSC5 protein was next validated by Western blot and IHC in Zhejiang cohort 2 and 3 (Figure 1D-1F).

As shown in Table 1, we investigate whether EXOSC5 expression was associated with the clinicopathological features and over survival. Upregulation of EXOSC5 was significantly correlated with tumor size and TNM stage. On the other hand, EXOSC5 was not significantly associated with the gender, age, Grade, tumor site, invasive depth, nodal metastasis, distant metastasis and TNM stage. Kaplan-Meier analysis revealed that GC patients with overexpression EXOSC5 had remarkably shorter overall survival (OS) compared to patients with low EXOSC5 expression in K-M plotter and our cohorts (Figure 1G). Cox Univariate and multivariate analysis indicated that EXOSC5 expression was an independent prognostic factor for OS (Table 2). Altogether, EXOSC5 is significantly upregulated in GC tissues and can be a potential oncogenic factor.

### Knockdown of EXOSC5 suppressed the proliferation and tumorigenesis of GC cells

**Figure 2:**
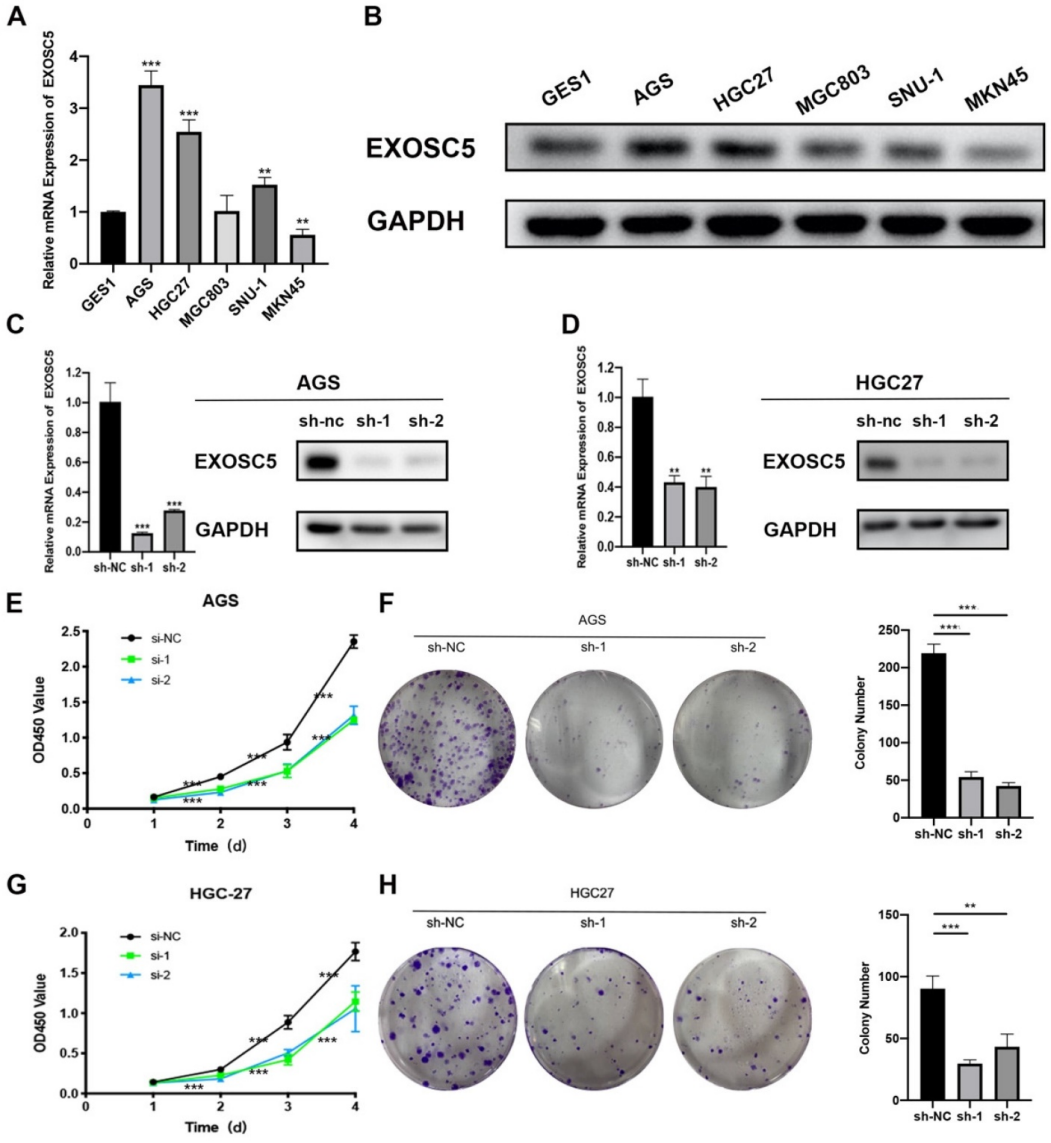
** Silenced EXOSC5 inhibits GC cells proliferation *in vitro*. (A, B)** mRNA and protein levels of EXOSC5 in GC cell lines and GES1. **(C, D)** The efficiency of EXOSC5 knockdown in AGS and HGC27 were determined by qPCR and Western blot. **(E-H)** CCK-8 assay and colony formation assay in AGS and HGC27 with shRNA/NC. Data are shown as the mean ± standard deviation. *P < 0.05, **P < 0.01, ***P < 0.001.

To explored the underlying biological effect of EXOSC5 in GC development, the expression of EXOSC5 in GC cell lines (AGS, HGC27, MGC803, MGC45, SNU-1) was detected by qRT-PCR and Western blot (Figure 2A, 2B). EXOSC5 protein and mRNA were remarkably increased in GC cell lined compared with the human gastric epithelial cell line GES1. To further demonstrate the function of EXOSC5 in GC cells, we picked AGS and HGC27 cell lines for studies. EXOSC5 expression in these two GC cell lines was knocked down by two independent lentiviral shRNAs aiming to different regions of EXOSC5 mRNA. The shRNA knockdown efficiency of EXOSC5 expression was validated by qRT-PCR and Western blot (Figures 2C, 2D). As expected, CCK-8 assays and colony formation demonstrated that stable knockdown of EXOSC5 obviously inhibited AGS and HGC27 cells proliferation *in vitro* (Figures 2E-2H).

### Overexpression of EXOSC5 promoted the growth of GC cells

**Figure 3:**
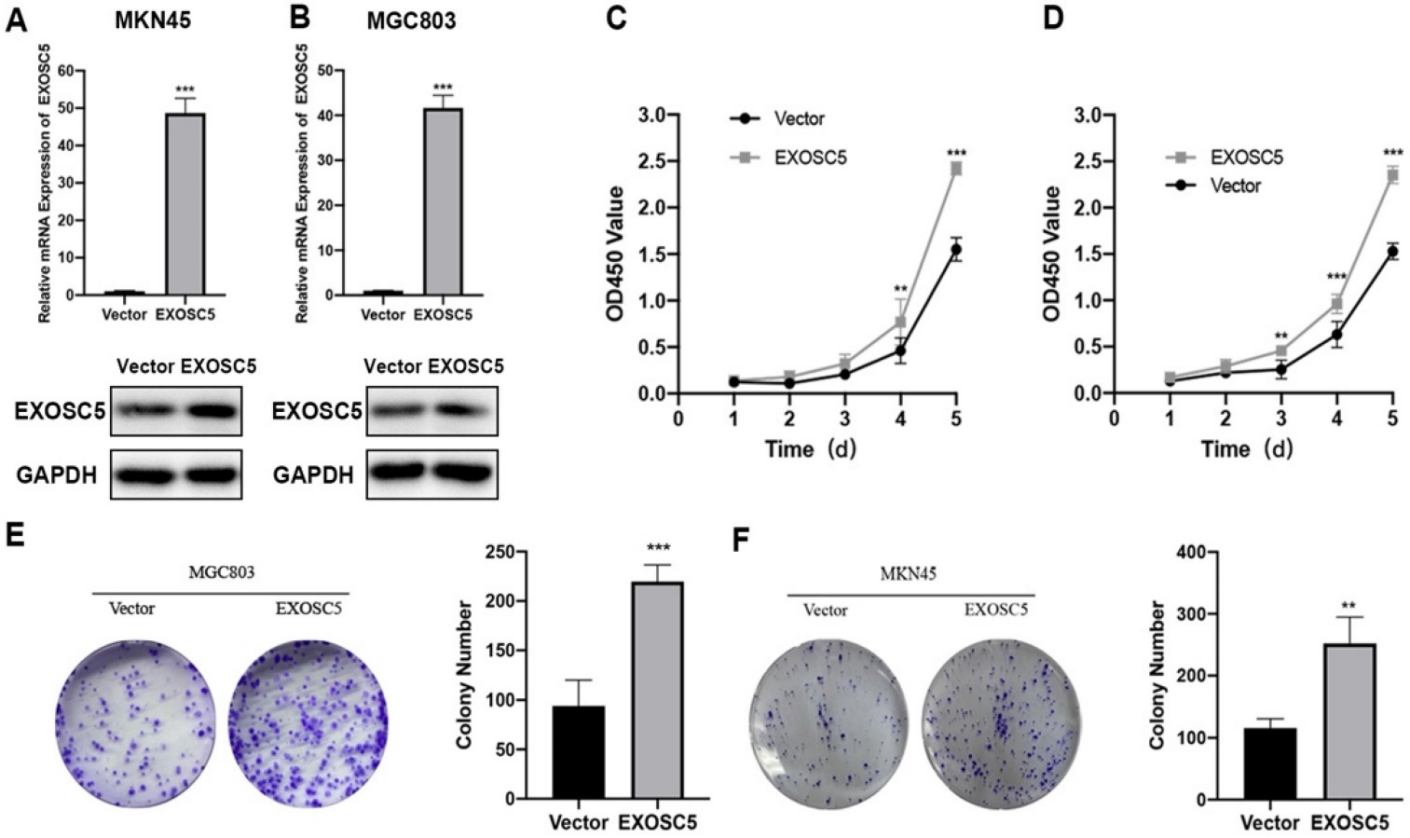
** Overexpression of EXOSC5 promoted the proliferation of GC and EXOSC5 knockdown suppresses G1/S transition of GC cells *in vitro*. (A, B)** The efficiency of EXOSC5 mRNA and protein expression levels of overexpressed EXOSC5 in MGC803 and MKN45 were determined by qPCR and Western blot. **(C-F)** CCK-8 assay and colony formation assay in MGC803 and MKN45 with EXOSC5 overexpression.

As shown above, EXOSC5 expression is relatively low in MCG803 and MKN45. Therefore, the MGC-803 and MKN45 cell lines of EXOSC5 overexpression were established, and the efficiency of EXOSC5 overexpression was confirmed by qRT-PCR and Western blot (Figure 3A, 3B). As analyzed by CCK8 assay, the cell proliferation rate of MGC803 and MKN45 was increased by EXOSC5 up-regulation (Figure 3C, 3D). Consistently, up-regulated EXOSC5 was profoundly increased colony formation in both cell lines (Figure 3E, 3F). Overall, these data demonstrated that EXOSC5 promoted GC cells proliferation *in vitro*.

### EXOSC5 knockdown led to G1 arrest in GC cells

**Figure 4:**
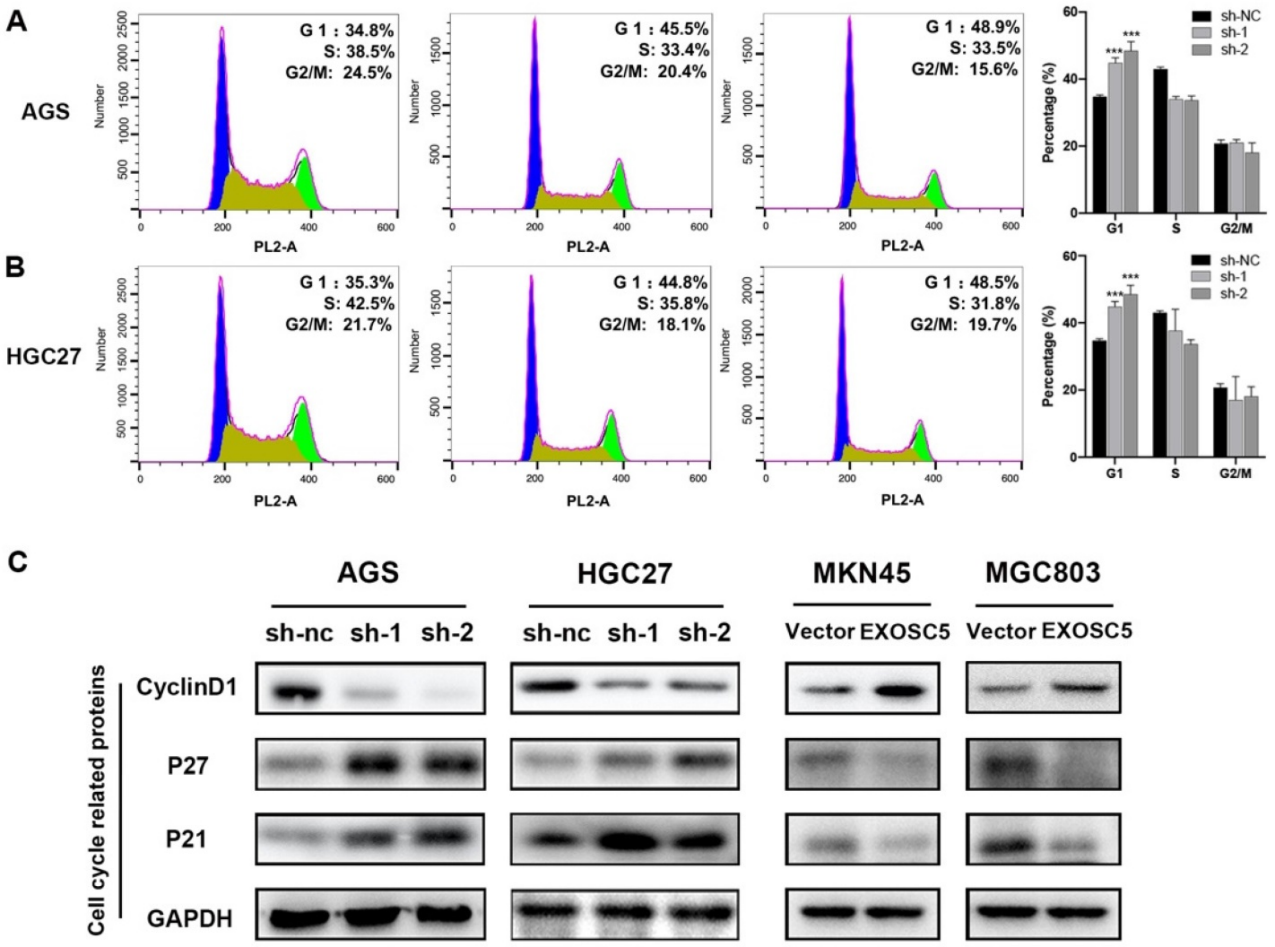
** EXOSC5 Knockdown led to G1 Arrest in GC. (A,B)** Flow cytometry showing the percentages of EXOSC5 knockdown cells and control cells at different cell cycle phase. **(C)** Cell cycle related proteins (P21, P27, Cyclin D1) in EXOSC5 knockdown and EXOSC5 overexpression cells by Western blot. Data are shown as the mean ± standard deviation. *P < 0.05, **P < 0.01, ***P < 0.001.

Our results showed that knockdown of EXOSC5 inhibited gastric cancer cell lines proliferation, so we hypothesized that EXOSC5 may be pivotal for cancer cell cycle regulation. To verify our hypothesis, flow cytometry was conducted to assess the transition in the cell cycle profile following EXOSC5 knockdown. The ratio of cells in the G1 phase was tremendously increased for depletion of EXOSC5 in AGS and HGC27, and the ratio of M phase cell population remarkably decreased (Figure 4A, 4B). Whereas, there was no difference for cells in the G2 phase after EXOSC5 knockdown. Our data indicated that EXOSC5 knockdown attenuates GC cells proliferation by inhibiting the G1/S transition. Next, the effects of EXOSC5 on various Cyclin-dependent kinases (CDKIs) and G1-S phase regulators cyclin D1, involved in cell cycle progression were assessed. The protein levels of p21 and p27 increased by EXOSC5 knockdown, while the cyclin D1 protein levels were significant decreased in AGS and HGC27. Conversely, these proteins showed opposite expression levels in EXOSC5 overexpression cell lines (MKN45 and MGC803) (Figure 4C). Therefore, EXOSC5 could control the G1/S transition to promote growth of CG cells through regulating cell cycle related proteins (P21, P27, Cyclin D1).

### AKT and STAT3 pathways are involved in EXOSC5-induced G1 arrest in GC cells

**Figure 5:**
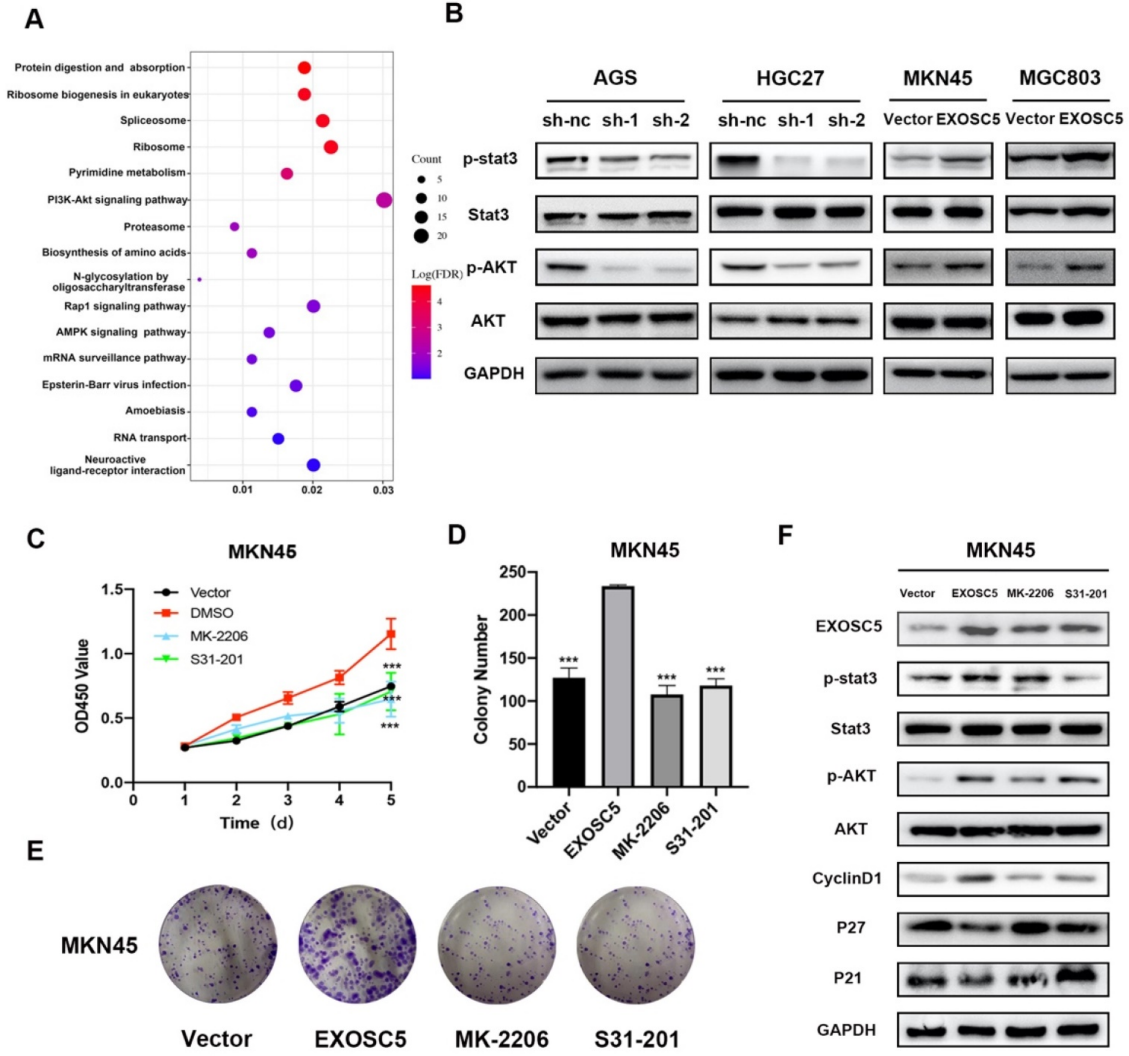
** EXOSC5 promote GC growth by AKT and STAT3 activation. (A)** KEGG pathway analysis of the genes significantly associated with the EXOSC5 expression in GC from cBioPortal and Coexpedia. **(B)** The protein expression levels of p-AKT, AKT, p-STAT3 and STAT3 in GC cell lines after EXOSC5 silencing or overexpressing. **(C, D)** Proliferation ability of MKN45 cells with EXOSC5 overexpression was determined by CCK8 and colony formation assay after treatment with MK-2206 (10 uM), S31-201 (10 uM) and DMSO. **(E)** The protein expression levels of p-AKT, AKT, p-STAT3, STAT3 and cell cycle related proteins (cyclin D1, p21 and p27) in MKN45 cells with EXOSC5 overexpression after treatment with MK-2206, S31-201 and DMSO. Data are shown as the mean ± standard deviation. ***P < 0.001.

To better understand the molecular mechanisms of EXOSC5-driven GC progression, we collected the overlapping genes between cBioPortal and Coexpedia to carry out GO and KEGG analyses in David v6.7 (http://david.abcc.ncifcrf.gov/) (Figure 5A). The KEGG analysis indicated that EXOSC5 was involved in AKT-associated signaling pathway. In addition, STAT3 pathways plays a vital role in the proliferation by regulating the expression of genes related to cell cycle in GC 15, 16. Interestingly, EXOSC5 knockdown inhibit phosphorylation of AKT and STAT3, while EXOSC5 overexpression contributed to the activation of AKT and STAT3 (Figure 5B), so these data demonstrated that AKT and STAT3 signaling pathways were involved in the progression of GC regulated by EXOSC5.

To further explore whether EXOSC5 could promote proliferation of GC via AKT and STAT3 signaling pathways, the EXOSC5 overexpression MKN45 were treated by the AKT inhibitor MK-2206 or STAT3 inhibitor S31-201. As shown in Figure 5C-5E, the abilities of proliferation and colony formation were significantly inhibited after the application of inhibitors. Additionally, the two inhibitors effectively decreased phosphorylation of AKT and STAT3 respectively, and rescued cell cycle related proteins (P21, P27, Cyclin D1) as compared to EXOSC5 overexpression MKN45 (Figure 5F). These results further confirmed that EXOSC5 enhanced GC cells growth via regulating AKT and STAT3-related signaling pathways.

### The roles of EXOSC5 in organoid of GC

**Figure 6:**
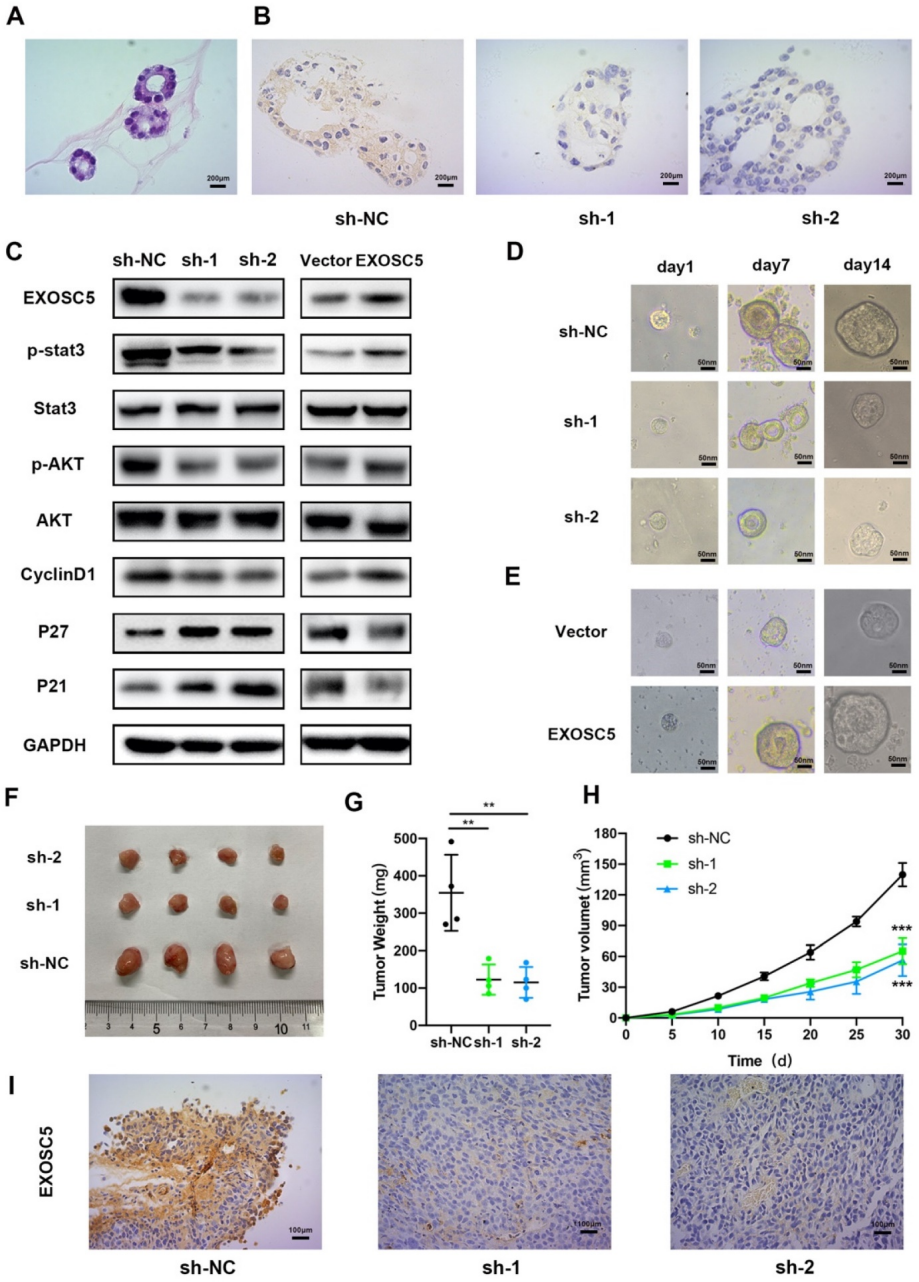
** EXOSC5 promote GC growth by AKT and STAT3 activation in GC organoid and vivo. (A)** Hematoxylin-eosin staining (H&E) of GC organoid (400x). **(B)** Immunohistochemistry (IHC) of GC organoid after EXOSC5 silencing. **(C)** The protein expression levels of p-AKT, AKT, p-STAT3, STAT3 and cell cycle related proteins (cyclin D1, p21 and p27) in GC organoid after EXOSC5 silencing or overexpressing. **(D, E)** The growth of organoid model with EXOSC5 knockdown or overexpression was assessed every 7 days. **(F)** Representative images of subcutaneous tumors in nude mice injected HGC27 cells transferred with shRNA. **(G, H)** Tumor volumes were measured by growth curve every 5 days and final weights of tumor were measured on the terminal days. **(I)** EXOSC5 staining in xenografted HGC27 tumors silencing EXOSC5. Data are shown as the mean ± standard deviation. **P < 0.01, ***P < 0.001.

Recent studies have demonstrated that organoids capture stably preserve genetic, proteomic, morphological and pharmacotypic features of the parent tumor *in vitro*, while can be used to model organ development to offer unprecedented genomic and environmental manipulation 17. In order to explore the biological functions of EXOSC5 in GC organoid, we built human GC organoid models from resected primary human GC tissues (Figure 6A), and knocked out EXOSC5 using shRNA. Compared with the control, shRNA suppression exhibited efficient knockout of EXOSC5 (Figure 6B), and EXOSC5 expression was comfirmed by Western blot (Figure 6C). During the sphere growth period, we observed that EXOSC5 knockdown significantly inhibited the growth of the organoids models (Figure 6D, Figure S1A), while EXOSC5-overexpressing GC organoid showed a significant increase in growth (Figure 6E, Figure S1B). We detected the downregulation of G1-S phase regulators cyclin D1 and upregulation of CDKIs (p21, p27) via inhibiting the phosphorylation of AKT and STAT3 in human GC organoid when we knocked down EXOSC5 (Figure 6C). Conversely, overexpression EXOSC5 reversed the expression levels of AKT, STAT3 and cell cycle related proteins (Figure 6C). These data indicate that the role of EXOSC5 in promoting proliferation also applies to GC organoid.

### EXOSC5 silencing inhibits tumor growth of GC *in vivo*

Tumorigenesis assays by subcutaneous injection with HGC27 cell lines stably expression scramble control and two EXOSC5 shRNAs were performed in nude mice, and tumor growth was monitored and measured every day. As can be seen in Figure 6F, the volumes and weights of tumor in the sh-EXOSC5 cell injection group were significantly smaller than the control group (Figure 6G, 6H). After sacrificing the mice, we detected that the EXOSC5 expression level was significant reduced in tumors from the sh-EXOSC5 mice group compared with that from the control group by IHC (Figure 6I). These findings supported the hypothesis that EXOSC5 deletion inhibited GC growth *in vivo*.

## Discussion

**Figure 7:**
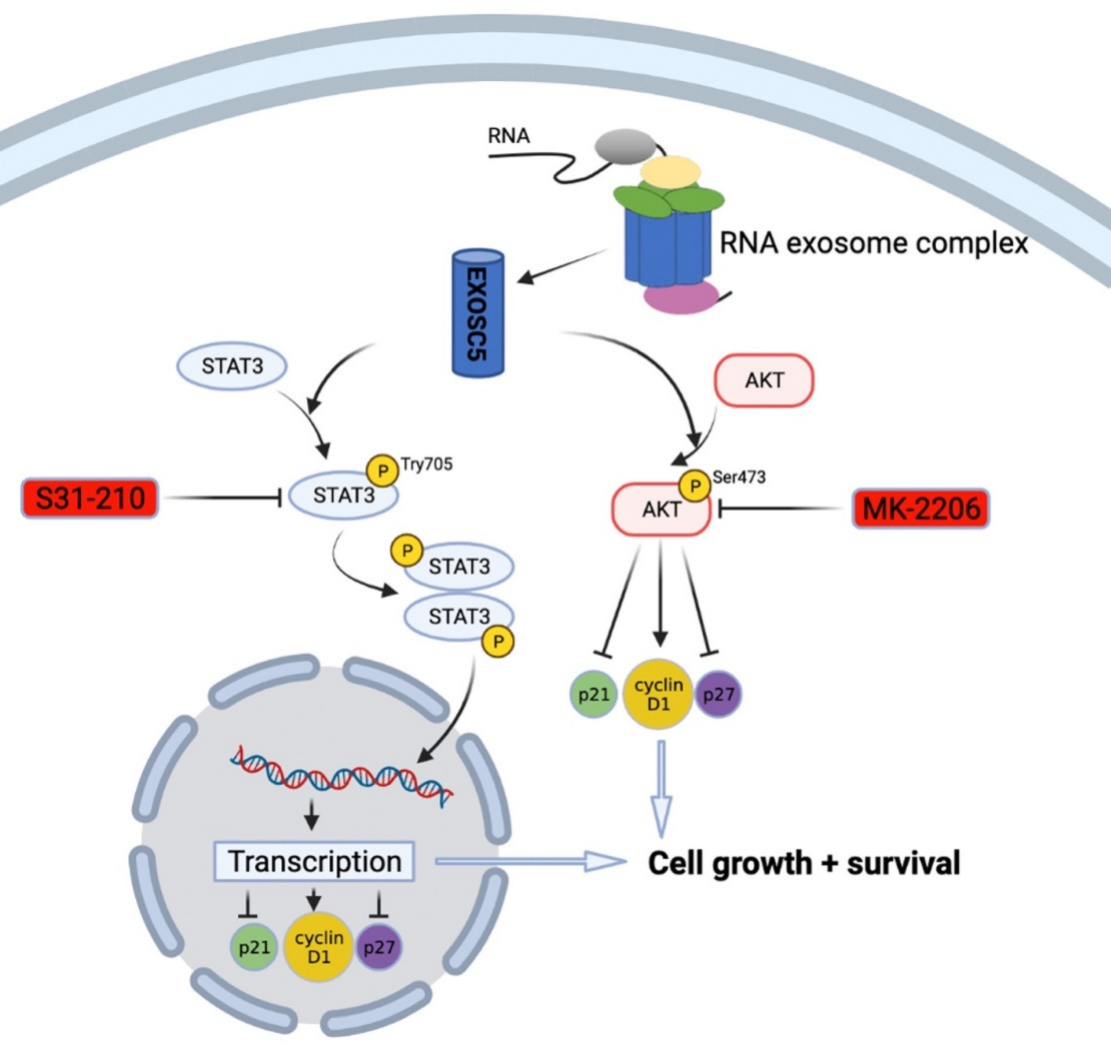
Schematic model of EXOSC5/AKT/STAT3 axis in GC. EXOSC5 promotes growth and survival in GC by regulating cell cycle proteins (P21, P27, Cyclin D1) via AKT and STAT3 pathways.

Previously, there was little known about the prognostic roles and molecular function of EXOSC5 in GC development. In this study, we used a straightforward approach to uncover the expression pattern and clinicopathological features of EXOSC5 in GC and its prognostic function with prognosis by integrative analysis of multiple cancer databases and clinical samples. Furthermore, functional and mechanism exploration revealed that knockdown of EXOSC5 significantly inhibited the growth of GC cells and targeted regulation of cell cycle factors cyclin D1, p21 and p27 via AKT and STAT3-associated signaling pathways (Figure 7). Additionally, we cultured organoid derived from fresh GC tissues to simulate relatively real GC environment, and provided evidence that EXOSC5 facilitates the proliferation of GC in organoids. Our results demonstrated that EXOSC5 functions as an oncogene to drive GC progression and therefore is a promising therapeutic target for the intervention of GC.

Although the expression of EXOSC5 has been studied in different cancers, there are limited reports about the clinical relevance and functional roles of EXOSC5 in solid tumors. Compared to normal tissues, increased expression of EXOSC5 was reported in many types of tumor cell lines, such as hematopoietic tumor, cervical carcinoma, melanoma, colon cancer cell and renal cell carcinoma 11, 18. Yang et al. purified recombinant EXOSC5 monoclonal antibody and found specific serological responses in 10-33% of patients with melanoma, lung cancer and prostate cancer. Owing to its expression and immunogenicity in a broad variety of malignancies, EXOSC5 deserve more evaluation as a target for tumor immunotherapy 11. Intriguingly, EXOSC5 were overexpressed in leukemic blasts from patients suffering from acute myelogenous leukemia and chronic myelogenous leukemia (CML) blast crisis but barely detectable in normal bone marrow, peripheral blood or leukemic cells from patients with stable-stage CML 19. Zhong et al. demonstrated that EXOSC5 and other eight genes forming Nine-RNA binding protein signature are served as prognostic genes to predict overall survival for kidney renal clear cell carcinoma 18. High expression of EXOSC5 is predictive of poor prognosis in colorectal cancer, and correlated with the tumor size. At the molecular mechanism, EXOSC5 plays a crucial part in growth of colorectal cancer via activating the ERK and AKT pathways 10. However, there are limited reports about the roles of EXOSC5 in GC. As far as we know, this is the first study to uncover the functional and clinical significance of EXOSC5 in GC by *in vivo* and *in vitro* experiments.

To test the roles of EXOSC5 in GC, we investigated the function of EXOSC5 in the regulation of GC cell proliferation using gain- and loss-of biological function assay. In this study, our findings indicated that EXOSC5 depletion suppressed proliferation by regulating G1/S transition, while opposite results were observed in EXOSC5 overexpressing GC cells. The cyclin-CDK complex cyclin D1 was widely reported to be an essential regulator of cell cycle process by inducing the transition from G1 to S phase 20. On the other hand, CDK inhibitors (p21 and p27) have been found to restrict transition in the cell cycle though restraining cyclin-CDK complexes 21. After knockdown EXOSC5, the expression of CyclinD1 was markedly reduced and p21/p27 levels were elevated. These data supported our study that EXOSC5 is an important contributor to GC progression by controlling cell cycle.

To investigate the molecular mechanisms of EXOSC5 in proliferation of GC, we found that AKT and STAT3 pathways might be involved in the stimulatory effects. The AKT and STAT3 signaling pathways are responsible for proliferation and cell cycle, and function as an oncogene in various types of tumors, including GC 22-24. Previous studies have demonstrated that AKT and STAT3 signaling pathways were positive regulator of cyclin D1 and negative regulators of p21 and p27 25, 26. In this study, we found that overexpression of EXOSC5 could activate AKT and STAT3 pathways, as evidenced by increased both proteins phosphorylation. At the same time, exogenous EXOSC5 could enhance of the phosphorylation of AKT and STAT3, and regulate cell cycle related proteins (cyclinD1, p21 and p27). These changes were verified by rescue assays in MKN45. Therefore, EXOSC5 promoted the proliferation in GC cells via AKT and STAT3-associated signaling pathways.

## Conclusions

In summary, this study reports an altered EXOSC5 expression pattern in GC and reveals its clinicopathologic relevance. Furthermore, functional and mechanistic studies suggested a crucial function of EXOSC5 in GC cell proliferation through the regulation of cell cycle related proteins cyclin D1, p21 and p27 partially via AKT and STAT3 signaling pathways. Therefore, we proved that EXOSC5 assumes the role of oncogene to promote the proliferation of GC cells and will be a promising therapeutic target in GC in the years to come.

## Supplementary Material

Supplementary figure.

## Figures and Tables

**Table 1:** Association between EXOSC5 expression and clinicopathological features of GC in 130 GC patients

Clinicopathological parameters	Number of cases	EXOSC5 expresssion		*P* value
		Low	High	
**Gender**				0.355
Male	91	26	55	
Female	39	12	37	
**Age**				0.788
<60	47	17	30	
≥60	83	32	51	
**Grade**				0.112
G1-2	14	8	6	
G3	116	41	75	
**Tumor site**				0.307
Cardia	39	18	21	
Body/antrum	91	32	59	
**Tumor size**				0.037
<5 cm	67	31	36	
≥5 cm	63	18	45	
**Invasive depth**				0.252
T1/T2	9	5	4	
T3/T4	121	44	77	
**Nodal metastasis**				0.077
N0/1	51	24	27	
N2/3	79	25	54	
**Distant metastasis**				0.914
Yes	5	2	3	
No	125	47	78	
**TNM stage**				0.036
I/II	25	14	11	
III/IV	105	35	70	

Note: EXOSC5 low expression is defined as IRS = 0-6, Person Chi-Square Tests.

**Table 2:** Univariate and multivariate analyses of survival of 130 patients with gastric cancer

	Univariate analysis			Multivariate analysis		
	HR	95%CI	P value	HR	95%CI	P value
Age	2.221	0.015-4.861	**0.046**	2.493	1.102-5.640	**0.028**
Gender	0.707	0.360-1.389	0.314	0.611	0.296-1.261	0.183
Location	1.44	1.222-6.336	0.291	1.236	0.610-2.503	0.557
Size	2.118	1.088-4.121	**0.027**	1.948	0.959-3.957	0.065
Differentiation	2.705	0.647-11.30	0.173	1.022	0.219-4.759	0.978
T stage	2.909	0.401-21.38	0.289	1.702	0.218-13.28	0.612
N stage	1.905	0.916-3.961	0.085	1.836	0.732-4.605	0.196
M stage	0.845	0.116-6.178	0.868	0.37	0.048-4.605	0.370
TNM	2.19	0.773-6.207	0.140	0.841	0.218-3.245	0.802
EXOSC5	2.782	1.222-6.336	**0.015**	2.421	1.029-6.640	**0.043**

## Abbreviations

GC: gastric cancer
EXOSC5: Exosome component 5
TCGA: the Cancer Genome Atlas
OS: Overall survival
IHC: Immunohistochemistry
H&E: Hematoxylin-eosin staining
CML: Chronic myelogenous leukemia
AJCC: American Joint Committee on Cancer
FBS: Feta bovine serum
SD: Standard deviation
