# Supplementary material for: EXOSC5 promotes proliferation of gastric cancer through regulating AKT/STAT3 signaling pathways
Source: J Cancer. 2022 Feb 28;13(5):1456–67. doi: 10.7150/jca.69166 (PMC8965127; doi:10.7150/jca.69166)
Supplement: Supplementary file 1 — Supplementary figure. [file jcav13p1456s1.pdf]

Supplementary Figures

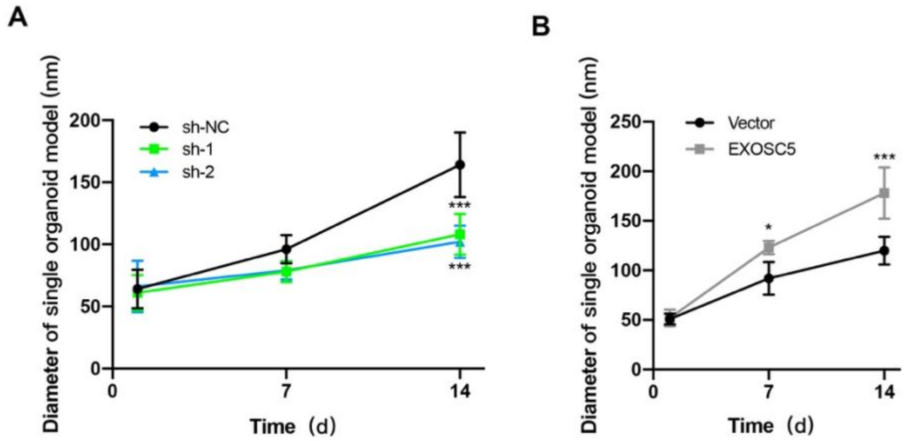

Figure S1. (A) The diameter of organoid were measured every 7 day after EXOSC5 knockdown. (B) The diameter of organoid were measured every 7 day after EXOSC5 overexpression. Data were expressed as mean ± standard deviation. *P < 0.05, **P < 0.01, ***P < 0.001.
